# Association Study of *PDCD1* Gene Variants and Its Gene Expression with Cutaneous Melanoma in a Mexican Population

**DOI:** 10.3390/genes16080866

**Published:** 2025-07-24

**Authors:** Fernando Valdez-Salazar, Luis A. Jiménez-Del Rio, Elizabeth Guevara-Gutiérrez, Andrea Melissa Mendoza-Ochoa, María José Zorrilla-Marina, Diana Karla García-Nuño, Jorge R. Padilla-Gutiérrez, José F. Muñoz-Valle, Emmanuel Valdés-Alvarado

**Affiliations:** 1 Instituto de Investigación en Ciencias Biomédicas (IICB), Centro Universitario de Ciencias de la Salud, Universidad de Guadalajara, Guadalajara 44340, Mexico; fernando.valdez9882@alumnos.udg.mx (F.V.-S.);; 2 Departamento de Biología Molecular y Genómica, Universidad de Guadalajara, Guadalajara 44340, Mexico; 3 Departamento de Dermatología, Instituto Dermatológico de Jalisco “Dr. José Barba Rubio”, Secretaría de Salud Jalisco, Zapopan 45190, Mexico; 4 Departamento de Dermatología, Hospital Civil de Guadalajara “Fray Antonio Alcalde”, Guadalajara 44200, Mexico

**Keywords:** melanoma, *PDCD1*, genetic variants, gene expression, qPCR, genetic association, Mexican population

## Abstract

**Background/Objectives**: Melanoma is an aggressive skin cancer influenced by genetic and immunological factors. The *PDCD1* gene encodes PD-1, a receptor involved in immune evasion and therapeutic response. This study aimed to evaluate the association of *PDCD1* variants (rs2227982, rs36084323, rs7421861) and its relative gene expression with melanoma in a Mexican population. **Methods**: An analytical cross-sectional study was conducted with 262 samples: 131 from melanoma patients (newly diagnosed and treatment-naïve) and 131 from cancer-free controls. Genotyping was performed using real-time PCR. *PDCD1* expression was assessed by qPCR, normalized with *GAPDH*, using the 2^−ΔΔCt^ method and the Pfaffl model. Statistical comparisons included allele/genotype frequencies, expression levels, and clinicopathological associations. **Results**: No significant association was found between the studied *PDCD1* variants and melanoma susceptibility. However, *PDCD1* was significantly overexpressed in melanoma samples (2.42-fold increase; *p* < 0.01), consistent across both quantification methods. Significant associations were also observed between histopathological subtype and Breslow thickness, and between subtype and anatomical site (*p* < 0.01). **Conclusions**: Although *PDCD1* variants showed no association with melanoma risk, the gene’s overexpression highlights its potential relevance in melanoma immunobiology. These findings contribute to the molecular characterization of melanoma in the Mexican population and support future research on *PDCD1* as an immunological biomarker.

## 1. Introduction

The skin, the largest organ of the human body, is composed of three primary layers: the epidermis, dermis, and hypodermis. Skin cancer, characterized by the uncontrolled growth of abnormal cells, originates in the epidermis, which is made up of squamous cells, basal cells, and melanocytes [1]. Repeated exposure of the skin to unprotected ultraviolet (UV) radiation from sunlight is a major contributing factor to the development of this type of cancer [2]. Melanoma is a form of skin cancer that arises from melanocytes, the cells responsible for skin pigmentation, and is associated with high mortality due to its rapid metastatic potential [3].

Although less common than other forms of skin cancer, such as basal cell carcinoma (BCC) and squamous cell carcinoma (SCC), melanoma accounts for approximately 4% of skin cancer cases but is responsible for most skin cancer-related deaths. Chronic and unprotected sun exposure is recognized as the principal risk factor for melanoma development; however, genetic predispositions also contribute to its occurrence [4]. Individuals with a family history of melanoma, fair skin, multiple atypical nevi, or a history of severe sunburns during childhood are at higher risk for this disease.

In this context, the *PDCD1* (*Programmed Cell Death 1*) gene, which encodes the PD-1 protein, plays a key role in regulating the immune response. PD-1 is involved in the inhibition of T cell activation, a critical process for preventing autoimmunity. However, in cancer, the interaction of PD-1 with its ligands PD-L1 and PD-L2 on tumor cells can allow immune evasion, thereby promoting tumor growth and dissemination [5]. Genetic variants in *PDCD1*, such as rs2227982 G>A, rs36084323 C>T, and rs7421861 A>G, have been associated with susceptibility to various types of cancer, including melanoma, by altering the function or expression of the PD-1 protein [6].

Melanoma has a high mortality rate due to its capacity to spread rapidly to other parts of the body. Therefore, identifying genetic factors that contribute to susceptibility is essential. In the Mexican population, melanoma has shown increasing incidence, particularly in the western region of the country. Studies by Gao et al. (2017) and Wagner et al. (2021) suggest that *PDCD1* variants may be involved in modulating the immune response in melanoma patients [6,7]. However, to date, little research has been conducted on *PDCD1* variants and their impact on melanoma in this population.

This study aims to analyze the association between the rs2227982 G>A, rs36084323 C>T, and rs7421861 A>G variants of the *PDCD1* gene, as well as its gene expression, with cutaneous melanoma in patients from the western region of Mexico. Therefore, this study aimed to evaluate the expression levels and genetic variability of *PDCD1* in a well-characterized Mexican population diagnosed with cutaneous melanoma, integrating both genotypic and gene expression analyses with clinical and histopathological variables. Unlike most studies that focus primarily on metastatic cases or therapeutic response, our approach explores *PDCD1* in a non-metastatic, treatment-naïve population, offering new insights into its potential role in disease susceptibility and early biological behavior. This integrative analysis in an underrepresented population adds a novel perspective to the current understanding of *PDCD1* in melanoma.

## 2. Materials and Methods

### 2.1. Study Design

The present study was designed to evaluate whether common genetic variants in the *PDCD1* gene, as well as its relative expression, are associated with cutaneous melanoma in a Mexican population. Specifically, we aimed to determine whether these variants were more frequent among cases versus controls, and whether they could be linked to molecular patterns or clinical characteristics of the disease. Given the immunomodulatory role of PD-1, identifying such associations could provide insights into the genetic predisposition to melanoma and inform future research on immune evasion mechanisms and potential predictive biomarkers for immunotherapy.

A cross-sectional analytical study was conducted to evaluate the association between the *PDCD1* gene variants rs2227982 G>A, rs36084323 C>T, and rs7421861 A>G and melanoma in patients from western Mexico. The analysis included both genotyping of the variants and quantification of *PDCD1* gene expression, as well as the association with patients’ clinical characteristics.

### 2.2. Study Population

The study population comprised patients diagnosed with cutaneous melanoma who were treated at the Instituto Dermatológico de Jalisco and Hospital Civil “Fray Antonio Alcalde” in Guadalajara, Jalisco. Patients were matched to a reference group (RG) of healthy individuals without a history of skin cancer.

All melanoma cases included were newly diagnosed and had not received any prior treatment at the time of sample collection. Given that the primary aim of the study was to assess constitutional genetic variants and gene expression in relation to disease susceptibility rather than treatment response or disease progression, clinical staging data were not incorporated into the analysis. Although limited information on disease stage was available for a small subset of patients, it was insufficient to ensure meaningful stratification and was therefore not considered in the present evaluation.

### 2.3. Inclusion and Exclusion Criteria

Inclusion criteria for the melanoma group were as follows:

- Patients diagnosed dermatoscopically and histologically with cutaneous melanoma.
- Patients over 18 years of age who voluntarily signed informed consent.
- Patients born in western Mexico (Jalisco, Nayarit, Michoacán, or Colima) with at least two previous generations (parents and grandparents) from those regions.

Exclusion criteria for both groups included:

- Individuals with a history of other cancers, transplant recipients, or immunosuppressed.
- Individuals who had received blood transfusions in the three months prior to sample collection.

Elimination criteria for both groups included:

- Incomplete molecular typing due to insufficient or degraded samples.
- Voluntary withdrawal from the study.

### 2.4. Sample Size

To estimate the required sample size for the genotyping analysis, we used OpenEpi version 3.01, applying the Fleiss method for proportions comparison. The calculation was based on the lowest expected allele frequency among the polymorphisms studied (rs2227982, minor allele frequency = 0.222), assuming an odds ratio of 2.0, a 95% confidence level, and 80% statistical power. The expected exposure proportion was 22.2% in controls and 36.3% in cases. Under these assumptions, a minimum of 88 individuals per group (176 alleles) was required. Our sample of 131 cases and 131 controls exceeded this threshold, supporting the robustness of the association analysis for *PDCD1* variants.

For the analysis of *PDCD1* relative gene expression, the minimum sample size was calculated using the standard formula for comparing means between two independent groups:

$$n=\frac{{2(Z\alpha +Z\beta )}^{2}S^{2}}{d^{2}}$$

where:

- *n* is the minimum number of individuals required per group;
- *Zα* = 1.96, is the corresponding to a 5% significance level;
- *Zβ* = 0.84, is thecorresponding to 80% statistical power;
- *S*^2^ = 1.56, is the estimated variance of ΔCt values; and
- *d* = 1.0, is the minimum difference to detect (equivalent to a 2-fold change in gene expression)

This resulted in a minimum requirement of 25 samples per group. In this study, 68 melanoma patient samples and 40 healthy controls were included, exceeding the minimum requirement, and ensuring adequate statistical power.

### 2.5. Molecular Analysis

An amount of 15 mL of blood was collected from each participant for DNA and RNA extraction. DNA was isolated from leukocytes using the modified Miller method and quantified using a NanoDrop Lite spectrophotometer. Genotyping of rs2227982 G>A, rs36084323 C>T, and rs7421861 A>G was performed by allelic discrimination using TaqMan^®^ probes on a LightCycler 96^®^ platform.

### 2.6. mRNA Expression

*PDCD1* expression was assessed by qPCR using *GAPDH* as the reference gene. RNA was extracted from leukocytes using TRIzol^®^ (Thermo Fisher, Waltham, MA, USA), quantified via spectrophotometry, and reverse-transcribed into cDNA using the High-Capacity cDNA Reverse Transcription Kit (Thermo Fisher, Waltham, MA, USA). qPCR amplification was conducted with specific primers for *PDCD1* and *GAPDH*. Results were expressed as relative expression of *PDCD1* normalized to *GAPDH*, using the 2^−ΔΔCt^ method.

### 2.7. Statistical Analysis

Clinical characteristics of melanoma patients were described by sex. Categorical variables were presented as absolute frequencies and percentages. Variant allele and genotype frequencies were compared between melanoma patients and controls using chi-square tests. Statistical analyses were performed in R (version 4.3.2).

### 2.8. Ethical Considerations

This study was approved by the Ethics Committee of the Centro Universitario de Ciencias de la Salud, Universidad de Guadalajara (approval code 23-59, CI-06623). All participants provided written informed consent. Biological samples and personal data were handled according to the Declaration of Helsinki and national and international regulations.

## 3. Results

### 3.1. Sociodemographic Characteristics

A total of 262 samples were analyzed, comprising 131 patients diagnosed with melanoma and 131 individuals in the reference group. The median age was 62 years (interquartile range: 51–73) in the melanoma group and 64 years (53–73) in the control group, with no statistically significant differences between groups (*p* = 0.75). Sex distribution was also similar, with a predominance of females in both groups (58% in melanoma vs. 52.7% in controls; *p* = 0.45).

Regarding skin phototype distribution, notable differences were observed between groups. Phototype III was the most frequent in the control group (53.4%), while the melanoma group showed a more heterogeneous distribution, with phototypes II (27.5%), III (30.5%), and IV (32%) being the most prevalent. Phototype I was observed exclusively among melanoma patients (*n* = 9), and phototype V appeared at low frequency in both groups (*n* = 3). Detailed sociodemographic characteristics are presented in Table 1.

### 3.2. Clinical Characteristics of Melanoma Patients

The clinical characteristics of melanoma patients, stratified by sex, are shown in Table 2. The most frequent anatomical location was the lower limbs, observed in 38.9% of women and 21.2% of men. In contrast, the head and neck region were more common among men (32.7%) than women (22.2%). Regarding histological subtype, superficial spreading melanoma was the most prevalent in both groups (44.1% in women and 39.6% in men), followed by nodular and lentigo malign subtypes.

With respect to tumor thickness measured by Breslow index, 35.8% of women presented lesions smaller than 1 mm, compared to 24.4% of men. A similar percentage of patients from both sexes showed melanomas with Breslow thickness > 4 mm (28.3% in women and 26.8% in men). In Clark’s classification, levels III and IV were the most frequently observed, with no notable differences between sexes. None of the comparisons showed statistically significant differences (*p* > 0.05).

### 3.3. Genotyping of PDCD1 Variants

Table 3 presents the genotyping results for the three *PDCD1* variants in melanoma patients and controls. For the rs2227982 G>A variant, the wild-type allele (G) was more frequent in both groups, accounting for 80.1% in melanoma patients and 77.8% in controls. The homozygous wild-type genotype (G/G) was also predominant, representing 64.1% of cases and 62.5% of controls. The odds ratio (OR), used to assess the association between genotypes and disease, indicated that the presence of the variant allele (A), both in heterozygous and homozygous form, was associated with a reduced risk of melanoma. However, this association did not reach statistical significance (*p* > 0.05). The Hardy–Weinberg equilibrium (HWE) test conducted in the control group yielded a *p* value of *0.42*, indicating no significant deviation in allele distribution and suggesting that the sample is in equilibrium.

For the rs36084323 C>T variant, the wild-type allele (C) was more frequent as well, observed in 80.5% of melanoma patients and 76.7% of controls. The C/C genotype was found in 65.6% of cases and 60.3% of controls. The OR analysis showed a similar trend, as with the previous variant. The calculated ORs of 0.83 for the heterozygous genotype and 0.61 for the homozygous variant genotype suggested a decreased risk of disease, but again, the results were not statistically significant. The Hardy–Weinberg test for this variant showed a *p* value of 0.64, confirming that the control group sample is in equilibrium.

Finally, genotyping of the rs7421861 A>G variant showed a predominance of the variant allele (G) in both allelic and genotypic distributions. The G/G and A/G genotypes were the most frequent in both groups. The association test between this variant and melanoma yielded OR values below 1, which would indicate a decreased risk if statistical significance had been achieved. However, this was not the case. The HWE test showed a *p* value of 0.85, confirming that the control group sample was in equilibrium for this variant as well.

### 3.4. Linkage Disequilibrium

**Figure 1:**
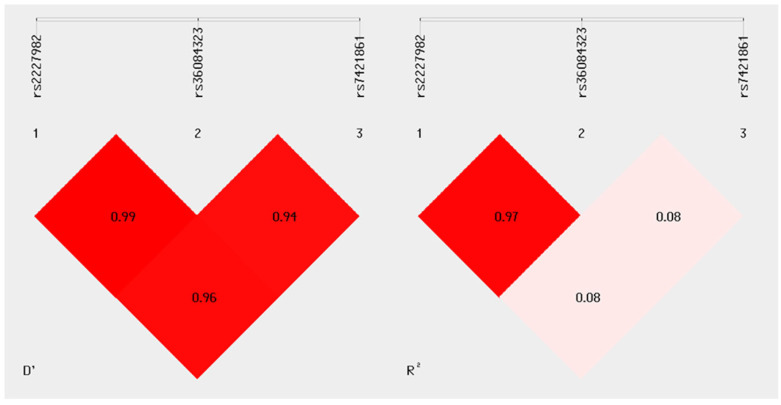
Linkage disequilibrium analysis. The graph was generated using SHEsis Plus software. The left panel shows the *D*’ values for each pair of variants, while the right panel displays the corresponding *R*^2^ values. A nearly complete LD (*D*’ > 0.94) is observed among the three analyzed polymorphisms, with a strong allelic correlation found only between rs2227982 and rs36084323 (*R*^2^ = 0.97), indicating a high likelihood of co-inheritance between these two alleles.

The results of the linkage disequilibrium (LD) analysis revealed high *D*’ values, particularly between variants rs2227982 and rs36084323 (*D*’ = 0.99), as well as between rs2227982 and rs7421861 (*D*’ = 0.96), indicating that these variant pairs are almost completely linked in the studied population (Figure 1). The relationship between rs36084323 and rs7421861 also showed a *D*’ of 0.94, suggesting strong co-segregation of these alleles in the sample.

However, when examining the *R*^2^ values, the correlation between rs2227982 and rs7421861, as well as between rs36084323 and rs7421861, was low (*R*^2^ = 0.08 for both pairs), suggesting that although these alleles tend to be inherited together, there is no strong predictive association in terms of allele frequency between them. In contrast, the rs2227982 and rs36084323 pair showed both a high *D*’ (0.99) and a high *R*^2^ (0.97), indicating not only linkage but also a strong correlation in allele frequency. This may reflect the joint inheritance of these alleles in haplotypic blocks within the analyzed population.

These LD findings are particularly relevant for evaluating the potential association of specific *PDCD1* variant combinations with differential susceptibility or protection to melanoma in the study sample.

### 3.5. Haplotypes

In addition to the analysis of individual variants, a haplotype analysis was conducted to identify allele combinations that might be associated with increased risk or protection against the studied phenotype. The haplotype results for the melanoma group are presented in Table 4.

Three main allele combinations were identified in the haplotype analysis for patients with melanoma: GCA, GCG, and ATA. The GCA haplotype was used as the reference, comprising the wild-type alleles at the three loci. The GCG and ATA haplotypes showed intermediate frequencies and were observed in both cases and controls, with no notable differences between groups. The calculated odds ratios for these haplotypes did not reveal statistically significant associations with melanoma presence.

It is important to note that haplotypes with a frequency below 0.03 were excluded from this analysis to avoid unstable and uninformative estimates.

### 3.6. Relative Expression of PDCD1

The relative expression of the *PDCD1* gene was assessed in samples from melanoma patients and controls. Quantitative PCR (qPCR) was performed, with normalization using the reference gene *GAPDH*.

**Figure 2:**
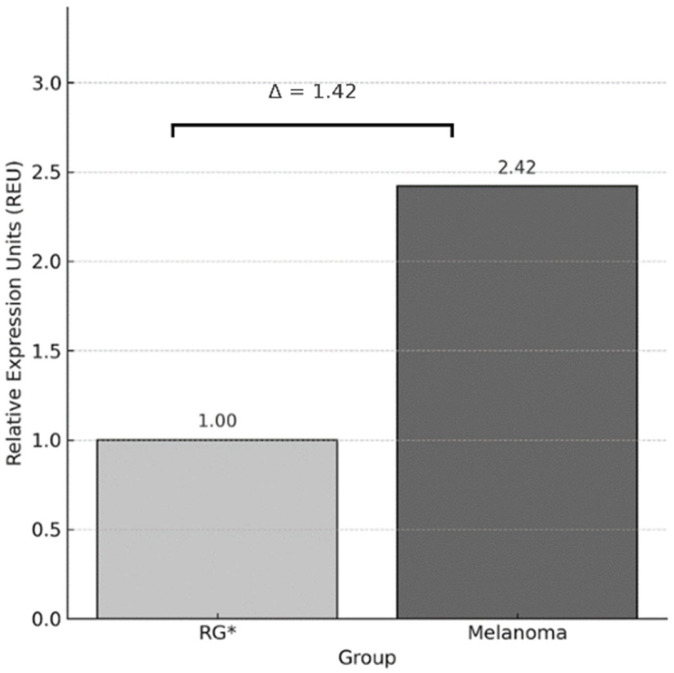
Relative expression of *PDCD1* using the Livak method (2^−ΔΔCt^). Bar graph showing the average Relative Expression Units (REU) of the *PDCD1* gene, normalized to *GAPDH,* and calibrated against the reference group (RG), which was assigned a value of 1. The melanoma group showed a 1.42-fold increase in *PDCD1* expression compared to RG. * RG: Reference Group.

The expression in melanoma patients was calculated using the 2^−ΔΔCt^ method, yielding a value of 2.42 (Figure 2). Considering that the reference group was calibrated to a value of 1, this result indicates a 1.42-fold increase in *PDCD1* expression in the melanoma group. Additionally, the Pfaffl method was used considering the amplification efficiencies (*PDCD1* = 2.04; *GAPDH* = 1.85) yielding a very similar result (2.42), which supports the consistency of the finding.

**Figure 3:**
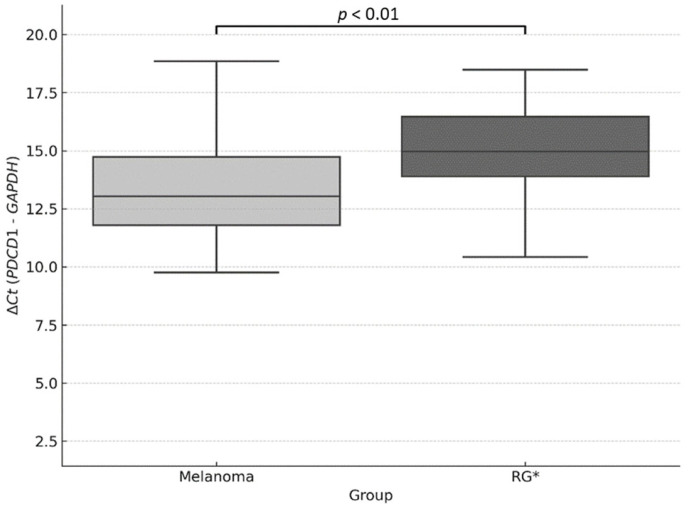
Relative quantification of *PDCD1* expression using the ΔCt method. Boxplot showing ΔCt values (*PDCD1*–*GAPDH*) in melanoma samples and the reference group (RG). Lower ΔCt values indicate higher relative gene expression. A statistically significant difference was observed between groups (*p* < 0.01; Mann–Whitney U test). * RG: Reference Group.

To validate the observed difference, a Mann–Whitney U test was performed on the individual ΔCt values, revealing a statistically significant difference between the two groups (*p* < 0.01), which supports the higher expression of *PDCD1* in the melanoma group (Figure 3).

### 3.7. Bivariate Analysis of Clinicopathological Characteristics

Bivariate analyses were performed using contingency tables and association tests (Fisher’s exact test or Chi-square test, as appropriate), aiming to identify statistically significant relationships among the clinicopathological characteristics of melanoma patients. All possible combinations between the variables included in the study were explored.

Among all pairwise comparisons, only two associations were found to be both statistically significant and clinically relevant: the relationship between histopathological subtype and Breslow thickness, and between histopathological subtype and anatomical location. Both associations showed *p* values < 0.01 (Table 5 and Table 6, respectively).

In the first association, superficial spreading melanomas were predominantly concentrated in lesions with lower depth (<2 mm), while nodular and acral lentiginous subtypes were more frequently associated with a Breslow index > 2 mm. Regarding anatomical distribution, lentigo maligna subtype occurred predominantly in the head/neck region, whereas the acral lentiginous subtype was almost exclusively located on the lower extremities. These distributions reveal distinctive clinicopathological patterns across melanoma subtypes.

Although these tables do not include genetic variables, they provide essential insights into the internal heterogeneity of melanoma, complementing the molecular findings and contributing to a more comprehensive understanding of the disease.

## 4. Discussion

Regarding sociodemographic characteristics, a median age of 62 years was observed among patients with melanoma, consistent with the findings of Lasithiotakis et al., who reported that most cutaneous melanoma cases occur in older adults, with a higher incidence in individuals over 55 years old, especially in men [8]. This pattern may be explained by cumulative ultraviolet radiation exposure throughout life, as well as factors related to immunosenescence.

In terms of sex distribution, a slight predominance of women (58%) was observed among melanoma cases, contrasting with global studies such as that of Whiteman et al., which reported a higher incidence in men, particularly in regions such as Oceania, Europe, and North America [9]. However, studies conducted in Latin America, such as that by Sánchez-Sánchez et al. in a Mexican population, have documented more balanced sex proportions or a slight female predominance [10]. This variability in sex distribution may be partly explained by differences in health-seeking behaviors and awareness of skin pathologies. In recent years, there has been a growing public awareness regarding skin cancer and the importance of early detection, particularly among women, who are more likely to seek dermatological evaluation due to aesthetic concerns and social norms. These factors could contribute to a higher detection rate in women and partially account for the observed sex-related differences across populations [11,12].

In our cohort, the most frequent histological subtype was superficial spreading melanoma (SSM), accounting for 37.4% of cases, followed by lentigo maligna (18.3%), acral lentiginous (16.0%), and nodular (16.8%). This distribution aligns with Caucasian populations where SSM predominates and benefits from earlier clinical detection due to its radial growth phase [13]. However, the relatively higher proportions of lentigo maligna and acral lentiginous melanoma in our sample may reflect several contextual factors. First, these subtypes often arise in chronically sun-exposed sites or anatomical areas prone to mechanical stress—lentigo maligna frequently appears in older adults on sun-damaged skin such as the face and neck, in part due to cumulative ultraviolet (UV) exposure and potentially limited sunscreen use [14]. Second, acral lentiginous melanoma (ALM) occurs on non-sun-exposed sites like palms and soles and is not strongly linked to UV exposure; mechanical trauma and increased melanocytic density in acral regions are implicated in its pathogenesis, which may explain its prominence in our population irrespective of sun protection habits [15].

Concerning anatomical location, lower limbs were the most common site (29.8%), especially among women (38.9%), consistent with Latin American studies reporting that women are more likely to develop melanomas on the legs, likely related to clothing choices, intermittent recreational sun exposure, and underuse of sunscreen on lower limbs [16]. In contrast, men more frequently presented lesions on the trunk and head/neck, which are typically exposed during occupational outdoor activities—studies have shown that occupational sun exposure increases melanoma risk in these regions, particularly at lower latitudes [17].

Analysis of Breslow thickness revealed that 49.5% of patients had melanomas thicker than 2 mm, indicating advanced-stage detection. This mirrors reports from Latin America, where delayed diagnosis—often due to limited access to dermatology specialists, lower use of sunscreen, and socioeconomic barriers contributes to elevated proportions of thick melanomas [18,19,20]. Importantly, the high proportion of ALM and lentigo maligna subtypes that are more likely to be diagnosed late also contributes to this trend. ALM, in particular, tends to present in later stages, as it occurs on less visible, non-sun-exposed surfaces, delaying detection [21,22].

In our study, no statistically significant associations were found between the *PDCD1* gene polymorphisms rs2227982, rs36084323, and rs7421861 and melanoma risk in our cohort. For *rs2227982*, the *G* and *A* alleles showed similar frequencies between the melanoma (80.1% and 19.8%, respectively) and control (77.8% and 22.1%, respectively) groups, with an odds ratio (OR) of 0.87 (95% CI:0.57–1.32; *p* = 0.51). At the genotypic level, the *G/A* genotype had an OR of 1.02 (95% CI:0.60–1.74; *p* = 0.92), and the *A/A* genotype had an OR of 0.54 (95% CI:0.17–1.68; *p* = 0.29), without significant differences.

These results contrast with previous studies that have reported significant associations between these polymorphisms and melanoma risk. For example, Boutros et al. found that the *G* allele of rs7421861 was associated with increased risk. In their study, the *G/G* genotype showed a relative risk (RR) of 1.65 (95% CI:1.32–2.05; *p* < 0.01) under the codominant model, and the *G* allele had an RR of 1.23 (95% CI:1.06–1.43; *p* < 0.01) under the allelic model [23].

Discrepancies between our findings and previous studies may be largely explained by differences in population genetic background, particularly given Mexico’s high level of admixture. The Mexican mestizo population from western regions—such as Jalisco—is composed of approximately 44–64% European, 30–39% Amerindian, and around 8% African ancestry [24]. Rangel-Villalobos et al. documented significant internal heterogeneity, with paternal European lineages predominant in the north and west and Amerindian ancestry more prevalent in the central and southern regions. Such regional and ancestral variation could influence allele frequencies of *PDCD1* polymorphisms, and consequently their association with melanoma risk. In fact, genetic associations identified in one population (e.g., European, or East Asian groups) cannot be directly extrapolated to admixed populations without adjustment for ancestry or population substructure [25]. Additionally, environmental exposures such as UV radiation intensity, sunscreen use, and occupational habits vary geographically and may interact with genetic predisposition, masking or unmasking genetic effects. Finally, differences in study design, genotyping platforms, and statistical models may further explain inconsistent associations in the literature [26,27].

In our study, the relative expression of *PDCD1* was significantly higher in melanoma patients compared to controls, with a 1.41-fold increase according to the 2^−ΔΔCt^ method and a 1. 42-fold increase using the Pfaffl method, considering specific amplification efficiencies for *PDCD1* and *GAPDH*. The Mann–Whitney U test applied to individual ΔCt values confirmed this difference with statistical significance (*p* < 0.01).

These findings are consistent with previous studies reporting overexpression of *PDCD1* in melanoma cells. For example, Holzgruber et al. showed that treating human melanoma cell lines A2058 and A375 with type I interferons (IFN-α or IFN-β) significantly induced *PDCD1* expression, both at the mRNA and surface protein levels, via the IFNAR-JAK-STAT1/2-IRF9 signaling pathway. This mechanism suggests that *PDCD1* overexpression in melanoma cells may be induced by tumor microenvironmental signals such as type I interferons [28].

That overexpression observed may reflect an immunosuppressive tumor microenvironment, consistent with the known inhibitory role of PD-1 in T cell activity. Several studies have shown that elevated *PDCD1* expression in tumors can be associated with immune evasion and poor prognosis in various cancers, including melanoma [29,30]. However, paradoxically, high *PDCD1* levels have also been linked to better responses to immune checkpoint inhibitors targeting the PD-1/PD-L1 axis [30]. Therefore, *PDCD1* overexpression might not carry a uniform prognostic meaning, but rather reflect the dynamic interplay between immune activation and inhibition in the tumor context.

Additionally, murine model studies have confirmed *PDCD1* expression in melanoma cells. A study using murine B16-F10 melanoma cells showed marked expression of *Pdcd1*, comparable to that of resting T cells and detectable by qPCR and flow cytometry. These findings support the notion that *PDCD1* expression is not limited to immune cells but may also be intrinsic to tumor cells [31].

Epigenetic regulation also plays a role in *PDCD1* expression. Studies have shown that global DNA hypomethylation in melanoma cells is associated with constitutive expression of immune evasion genes, including *PDCD1* [32]. Several studies have highlighted that the expression of *PDCD1* is not only regulated at the genetic level, but also through epigenetic mechanisms that may influence tumor behavior and prognosis. Röver et al. (2018) demonstrated that *PDCD1* promoter methylation is a prognostic factor in patients with lower-grade gliomas harboring *IDH* mutations, suggesting that epigenetic silencing of *PDCD1* could modulate immune evasion in the tumor microenvironment [33]. Similarly, Ricci et al. (2019) reported that methylation of the *PDCD1* promoter in Merkel cell carcinoma is significantly associated with tumor aggressiveness and clinico-pathological parameters, reinforcing its potential relevance as a prognostic biomarker [34]. More recently, Zhang et al. (2025) employed PD-1-targeted nanocarriers to enhance the delivery of curcumol in prostate cancer models, underscoring the importance of *PDCD1* not only in immune modulation but also in targeted therapeutic strategies [35].

The regulation of *PDCD1* expression and its impact on melanoma cannot be understood in isolation from the tumor microenvironment (TME), which orchestrates immune responses through a dense regulatory network. In melanoma, cytokines such as IFN-α/β can induce PD-1 expression directly on tumor cells via STAT1/2-mediated chromatin remodeling, highlighting the role of inflammatory signaling in enhancing PD-1 levels independently of T cell activation [36]. Additionally, an immunosuppressive TME—characterized by the accumulation of regulatory T cells, myeloid-derived suppressor cells, and tumor-associated macrophages—leverages the PD-1/PD-L1 axis to dampen antitumor immunity, facilitating disease progression and therapeutic resistance [37]. Moreover, metabolite accumulation such as lactic acid in glycolytic TMEs further promotes PD-1 expression on immune subsets, amplifying immune suppression within the tumor [38]. Hence, the elevated *PDCD1* expression observed in our cohort reflects not only gene regulation but also the dynamic interplay of immune and metabolic factors within the TME. Understanding this complexity is essential for interpreting *PDCD1*’s role in melanoma and designing future immunotherapeutic strategies.

In the bivariate analysis of clinic-pathological characteristics in our melanoma cohort, two statistically significant and clinically relevant associations were identified: the relationship between histopathological subtype and Breslow index, and the relationship between histopathological subtype and anatomical location, both with *p* < 0.01.

The first association showed that superficial spreading melanomas were concentrated in lesions of lower depth (<2 mm), while nodular and acral lentiginous subtypes were more frequently associated with a Breslow index > 2 mm. This observation aligns with previous studies showing that nodular melanomas tend to exhibit more pronounced vertical growth, resulting in deeper lesions and a worse prognosis. For instance, a study by López-Pardo Rico et al. found that nodular melanomas had greater Breslow thickness compared to other subtypes, translating into later detection and poorer outcomes [39].

Regarding the second association, lentigo maligna was predominantly located in the head/neck region, whereas acral lentiginous melanoma was almost exclusively found on the lower extremities. This anatomical distribution is consistent with the existing literature, which describes lentigo maligna as a common variant in chronically sun-exposed areas, such as the face and neck, especially in elderly patients [40]. In contrast, acral lentiginous melanoma is more common on the palms, soles, and subungual regions, and is more prevalent in darker-skinned populations.

Furthermore, studies have shown that the anatomical location and the histological subtype of melanoma are interrelated and can influence patient prognosis. For example, a study by Dessinioti et al. found that head-located melanomas were associated with higher recurrence risk, and certain histological subtypes, such as acral lentiginous, had higher recurrence rates than other subtypes [41].

The observed overexpression of PDCD1 in melanoma patients contributes to the understanding of the complex and multifactorial nature of melanoma, although no significant clinical associations with the analyzed genetic variants were identified in this study. The Breslow index is classically recognized as the most robust prognostic indicator, with each additional millimeter nearly doubling the risk of melanoma-related mortality [42,43,44]. In line with this, we found that nodular and acral lentiginous melanomas exhibited Breslow depths greater than 2 mm (*p* < 0.01), consistent with the literature reporting poorer outcomes for these subtypes compared to superficial melanomas, which generally have thinner lesions [45].

Heterogeneity in findings across previous studies may be explained by differences in environmental exposures (UV radiation, skin microenvironment), population genetic structure (allelic and mutational frequencies), and analytical methods. For example, acral melanomas, prevalent in darker-skinned populations, exhibit less UV exposure but worse outcomes and distinct genetic profiles [46,47]. Likewise, the techniques used to quantify gene expression (qPCR vs. RNA-Seq vs. immunohistochemistry) and amplification efficiency considerations may influence the magnitude of observed differences [27,48].

In a therapeutic context, the presence of germline variants in *PDCD1* or tumor overexpression of the receptor may predict responses to inhibitors such as nivolumab or pembrolizumab. For instance, patients with high intratumoral PD-1 expression showed better clinical responses to anti-PD-1 agents, whereas those with certain germline genetic variants derived less benefit [49,50]. Furthermore, epigenetic regulation (promoter hypomethylation) and microenvironmental signals (type I IFNs) may reflect tumor states that are more susceptible or resistant to immunotherapy [28,32].

## 5. Conclusions

In conclusion, no statistically significant associations were observed between the *PDCD1* variants rs2227982 G>A, rs36084323 C>T, and rs7421861 A>G and the presence of melanoma in the analyzed population. Nonetheless, a higher expression level of *PDCD1* was identified in individuals with melanoma, suggesting a potential immunological involvement of this gene in the context of the disease. These results contribute to the molecular profiling of melanoma in western Mexico and may support future research exploring *PDCD1* as an immunological marker.

## Figures and Tables

**Table 1:** Sociodemographic characteristics of the study groups.

Characteristic	Melanoma	Reference Group	*p*
*n*	131	131	
Age	62 (51–73)	64 (53–73)	0.75
Sex *n* (%)			0.45
Female	76 (58)	69 (52.7)	
Male	55 (42)	62 (47.3)	
Skin phototype			
I	9	0	
II	36	12	
III	40	70	
IV	42	42	
V	3	3	

Note: Totals may vary due to missing data.

**Table 2:** Clinical characteristics of patients with melanoma.

Characteristic	Level	Female *n* (%)	Male *n* (%)	Total *n* (%)	*p*
Anatomical location	Head/neck	16 (22.2)	17 (32.7)	33 (25.2)	0.14
	Trunk	13 (18.1)	14 (26.9)	27 (20.6)	
	Upper limbs	15 (20.8)	10 (19.2)	25 (19.1)	
	Lower limbs	28 (38.9)	11 (21.2)	39 (29.8)	
Histological subtype	Superficial spreading	30 (44.1)	19 (39.6)	49 (37.4)	0.90
	Nodular	12 (17.6)	10 (20.8)	22 (16.8)	
	Acral lentiginous	13 (19.1)	8 (16.7)	21 (16.0)	
	Lentigo maligna	13 (19.1)	11 (22.9)	24 (18.3)	
Breslow thickness	<1 mm	19 (35.8)	10 (24.4)	29 (22.1)	0.35
	1.1–2 mm	7 (13.2)	11 (26.8)	18 (13.7)	
	2.1–4 mm	12 (22.6)	9 (22.0)	21 (16.0)	
	>4 mm	15 (28.3)	11 (26.8)	26 (19.8)	
Clark level	I	4 (9.3)	6 (15.4)	10 (7.6)	0.77
	II	10 (23.3)	12 (30.8)	22 (16.8)	
	III	15 (34.9)	11 (28.2)	26 (19.8)	
	IV	12 (27.9)	8 (20.5)	20 (15.3)	
	V	2 (4.7)	2 (5.1)	4 (3.1)	

**Table 3:** Allelic and genotypic frequencies in the melanoma and reference groups.

	Melanoma *n* = 131 (%)	Reference Group *n* = 131 (%)	OR (CI 95%)	*p*
**rs2227982 G>A**				
Alleles				
G *	210 (80.1)	204 (77.8)	1	-
A	52 (19.8)	58 (22.1)	0.87 (0.57–1.32)	0.51
Genotypes				
G/G *	84 (64.1)	82 (62.5)	1	-
G/A	42 (32)	40 (30.5)	1.02 (0.60–1.74)	0.92
A/A	5 (3.9)	9 (7)	0.54 (0.17–1.68)	0.29
HWE				0.42
**rs36084323 C>T**				
Alleles				
C *	211 (80.5)	201 (76.7)	1	-
T	51 (19.4)	61 (23.2)	0.79 (0.52–1.21)	0.28
Genotypes				
C/C *	86 (65.6)	79 (60.3)	1	-
C/T	39 (29.7)	43 (32.8)	0.83 (0.49–1.31)	0.49
T/T	6 (4.7)	9 (6.9)	0.61 (0.20–1.79)	0.37
HWE				0.64
**rs7421861 A>G**				
Alleles				
A *	67 (25.5)	60 (22.9)	1	-
G	195 (74.4)	202 (77.1)	1.15 (0.77–1.72)	0.47
Genotypes				
A/A *	12 (9.1)	8 (6.1)	1	-
A/G	43 (32.8)	44 (33.5)	0.65 (0.24–1.75)	0.39
G/G	76 (58.1)	79 (60.4)	0.64 (0.24–1.65)	0.35
HWE				0.85

Note: OR = odds ratio; CI = confidence interval; HWE = Hardy–Weinberg equilibrium. The allele and genotype marked with an asterisk (*) represent the reference category.

**Table 4:** Haplotype frequencies in melanoma and reference groups, and their association with disease status.

Haplotype	Melanoma	Reference Group	OR (CI 95%)	*p*
GCA *	145 (0.553)	144 (0.549)	1	-
GCG	62 (0.236)	57 (0.217)	1.08 (0.70–1.65)	0.72
ATA	47 (0.179)	58 (0.221)	0.80 (0.51–1.26)	0.34

Note: OR = odds ratio; CI = confidence interval. The haplotype marked with an asterisk (*) represents the reference used in the association analysis.

**Table 5:** Association between melanoma histopathological subtype and Breslow thickness.

Breslow Thickness	Superficial Spreading	Nodular	Acral Lentiginous	Lentigo Maligna	*p*
<1 mm	16	1	4	8	<0.01
1.1 mm–2 mm	15	0	2	0	
2.1 mm–4 mm	6	7	5	3	
>4 mm	8	13	5	0	

Note: The association between histopathological subtype and Breslow thickness was assessed using Fisher’s exact test due to expected cell counts < 5 in multiple categories. A *p* value < 0.01 indicates a statistically significant relationship. All categories are shown to preserve the observed clinical distribution.

**Table 6:** Association between melanoma histopathological subtype and anatomical location.

Anatomical Location	Superficial Spreading	Nodular	Acral Lentiginous	Lentigo Maligna	*p*
Head/Neck	10	4	0	17	<0.01
Trunk	14	7	0	3	
Upper limbs	12	5	5	1	
Lower limbs	13	5	14	3	

Note: The association between histopathological subtype and anatomical location was assessed using Fisher’s exact test due to low expected counts in some categories. A *p* value < 0.01 indicates a statistically significant association.

## Data Availability

The original contributions presented in the study are included in the article; further inquiries can be directed to the corresponding author.
